# Supplementary material for: Risk Stratification of Patients with Pulmonary Arterial Hypertension: The Role of Echocardiography
Source: J Clin Med. 2022 Jul 12;11(14):4034. doi: 10.3390/jcm11144034 (PMC9323074; doi:10.3390/jcm11144034)
Supplement: Supplementary file 1 [file jcm-11-04034-s001.zip › jcm-1770247-supplementary.pdf]

Table S1. Patients Included in Study

Study	Enrollment Period	Total Number of PAH Patients with Echocardiographic Data	Number of PAH Patients Excluded from This Study	Number of PAH Patients Included in This Study
**Forfia et al. 2006 [13]**	2004	N = 47	N = 15 (Incident Patients)	N = 32
**Mathai et al. 2011 [17]**	2004–2009	N = 50	N = 37 (25 Incident Patients; 12 Prevalent Patients Already Included from Forfia et al.)	N = 13
**Mazurek et al. 2017 [18]**	2007–2013	N = 70	N = 0	N = 70
**Mukherjee et al. 2017 [15]**	2007–2015	N = 55	N = 24 (18 Incident Patients; 6 Prevalent Patients Already Included from Mathai et al.)	N = 31
**All Studies Combined**	2004–2015	N = 222	N = 76	N = 146

Abbreviations: PAH = Pulmonary Arterial Hypertension
